# Confirmatory factor analysis of the Patient Health Questionnaire-9: A study amongst tuberculosis patients in the Free State province

**DOI:** 10.4102/sajid.v35i1.242

**Published:** 2020-12-21

**Authors:** Gladys Kigozi

**Affiliations:** 1 Department Center for Health Systems Research & Development, Faculty Humanities, Free State University, Bloemfontein, South Africa

**Keywords:** tuberculosis, depression, PHQ-9, construct validity, confirmatory factor analysis

## Abstract

**Background:**

There is growing evidence that depression is frequently comorbid with tuberculosis (TB) and is often associated with a decreased quality of life and poor treatment outcomes. The Patient Health Questionnaire (PHQ-9) is widely used to screen for depression in clinical settings in low-and middle-income countries. This study examined the construct validity and reliability of an interviewer-administered PHQ-9 in a sample of new TB patients in the Free State province of South Africa.

**Methods:**

A pilot study was conducted in 2019 amongst 208 new adult TB patients attending primary healthcare facilities in the Lejweleputswa District in the Free State. Trained fieldworkers administered a structured questionnaire comprising of questions on patient’s socio-demographic characteristics and the nine-item PHQ-9 to the patients. Confirmatory factor analysis and Cronbach’s alpha were respectively used to investigate the construct validity and internal consistency of the PHQ-9.

**Results:**

The model was a good fit, with a Tucker–Lewis index of 0.976, a comparative fit index of 0.982 and a root mean square error of approximation of 0.062 (90% CI: 0.032–0.089). All indicators showed significant positive factor loadings, with standardised coefficients ranging from 0.467 to 0.799. The PHQ-9 was characterised by a single factor latent structure – depression – underlying all items. The Cronbach’s alpha of the scale was 0.84.

**Conclusion:**

The results support a unidimensional structure of the PHQ-9, with satisfactory internal consistency implying that the scale is valid and reliable. The TB programme can confidently consider the PHQ-9 for the routine assessment of depression amongst TB patients in the Free State province and similar settings.

## Background

Depression ranks amongst the leading causes of ill-health and disability worldwide.^1,2,3^ In 2017, depressive disorders were ranked 11th out of 30 top causes of disability-adjusted life years (DALYs).^2^ From a recent World Health Organization (WHO) report, 4.4% of the global population was estimated to suffer from depression in 2017.^3^ The same report indicated that the prevalence rate of depressive disorders in South Africa was 4.6%, contributing 7.2% to the total DALYs.^1^ Worryingly, in South Africa, a significant proportion of those with common mental health problems such as depression do not receive the necessary treatment and care.^4,5,6^ This is partially attributed to the poor integration of mental health services within primary healthcare (PHC), resulting in the non-recognition of mental illness as an underlying reason for many PHC complaints.^7,8^

There is growing evidence that depression is frequently comorbid with tuberculosis (TB) and is often associated with low quality of life, increased side effects, poor treatment adherence and outcomes, and increased mortality.^9,10,11,12,13,14,15,16^ A systematic review of 31 studies across 11 countries established that the prevalence rate of depression amongst TB patients ranged from 11.3% to 80.2%.^10^ A study conducted in Turkey established that depression was more common in TB patients who had defaulted and those suffering from multidrug-resistant TB (MDR-TB) compared with recently diagnosed patients.^17^ In Ethiopia, a study reported that tuberculosis–human immunodeficiency virus (TB–HIV) co-infected patients had a significantly greater risk for common mental disorders than their mono-infected counterparts.^9^ Whilst screening for depression in all TB patients is strongly recommended to ensure early diagnosis and treatment, a key challenge for TB programmes is how best to implement this intervention strategy.^14,18^

A global survey amongst National TB Programmes (NTPs) across 26 countries, including South Africa, revealed that only two NTPs incorporated routine screening for any mental disorder, only four assessed alcohol or drug use, and only five had standard protocols for the co-management of disorders. Whilst most NTP directors were receptive to the notion of integrated TB and mental healthcare, the main perceived barriers to service integration were limited capacity, non-recognition of mental health as a problem, insufficient resources and TB-related social stigma.^19^ Furthermore, in settings such as South Africa where facilities are typically high-burdened, with concomitant shortages of professional health workers, the routine screening of TB patients for depression necessitates easy-to-use, reliable, valid and context-specific screening tools.^20^ Such tools also need to accommodate use by non-mental health specialists.^8,21^

The Patient Health Questionnaire (PHQ-9) developed by Kroenke and colleagues^22^ has been widely used to screen for depression in clinical settings in low- and middle-income countries.^23^ The tool is useful for criterion-based diagnosis of depressive disorders and the assessment of the severity of depression.^22^ The PHQ-9 has been validated for use in various healthcare settings across Africa.^24,25,26^ In South Africa, the criterion validity of the PHQ-9 has been demonstrated amongst patients attending PHC in the North West and Gauteng provinces.^26,27^ The tool has also been translated and administered to patients proficient in at least 5 out of the 11 official languages in South Africa^26,27^ but it has never been translated to Sesotho for use in the Free State province.

There are mixed findings regarding the structure of the PHQ-9. Using exploratory factor analysis (EFA), previous studies in clinical settings in Africa^8,12,28^ have established a unidimensional structure of the PHQ-9, that is, depression. However, other studies in clinical settings in the developed countries have established that the PHQ-9 can display a bidimensional factor structure, that is, somatic and cognitive-affective factors.^29,30^ For this reason, the current study aimed to investigate the construct validity of an interviewer-administered PHQ-9 using confirmatory factor analysis (CFA) in a sample of TB patients. The hypothesis was that the unidimensional structure of the PHQ-9 as presented in other African settings would be replicated amongst TB patients in the Free State province of South Africa.

## Methods

### Design and setting

A pilot study was conducted amongst new TB patients across 11 PHC facilities in the Lejweleputswa District in the Free State. Data were gathered between November and December 2019. The clinics were purposefully selected based on high volumes of drug-susceptible TB patients.

### Participant sampling and recruitment

The study population comprised new adult patients susceptible to TB treatment. Patients were purposefully selected for the study. The patients were included in the study if they were 18 years and older, initiated treatment between 01 May 2019 and 31 October 2019 and were proficient in either Sesotho or English. Patients younger than 18 years, those who were too ill to be interviewed, those undergoing TB re-treatment and those with MDR-TB were excluded.

Eligible patients were recruited through their attending nurses. The nurses informed them about the study and referred them to trained fieldworkers located in private spaces within the facility premises. Those who were willing to participate in the study provided written informed consent for the interviews and access to their clinical information.

### Data collection

A structured questionnaire was used for data gathering. It comprised questions about patient socio-demographics and the nine-item PHQ-9.^22^ The questionnaire and consent forms were forward-translated into Sesotho and back-translated to English by two independent translators. The translators then reconciled the final translated draft by comparing the original questionnaire to the back-translated questionnaire. The process yielded minor discrepancies that were discussed with the research team before consensus was reached on the final translated questionnaire. A team of experienced bilingual fieldworkers administered the questionnaires to the patients through face-to-face interviews. On average, the questionnaire took about 10 minutes to complete.

### Measures

The PHQ-9 questionnaire is widely used to measure depression. It comprises items assessing the frequency of depressed mood over 2 weeks preceding assessment.^22^ Accordingly, patients in this study were asked to indicate how often they experienced the following: (1) little interest or pleasure in doing things (anhedonia), (2) depressed mood or hopelessness, (3) trouble falling asleep (insomnia) or sleeping too much (hypersomnia), (4) fatigue or loss of energy, (5) appetite disturbances, (6) feelings of guilt or worthlessness, (7) diminished ability to think or concentrate (cognitive dysfunction), (8) moving/speaking slowly [retardation] or being fidgety/restlessness [psychomotor agitation] and (9) suicidal ideation. Response options were on a 4-point Likert scale as follows: 0 = not at all, 1 = several days, 2 = more than half the days and 3 = nearly every day. In line with three validation studies in Africa,^24,26,27^ the response set was adapted to improve respondent comprehension, such that ‘several days’ was illustrated as 1–7 days; ‘half the days’ was illustrated as 8–11 days; and ‘nearly every day’ was illustrated as 12–14 days. Socio-demographic and clinical measures included sex (male or female), age (continuous variable), marital status (married or unmarried), education qualification (no formal or primary or secondary or tertiary) and HIV status (negative or positive or not recorded).

### Analysis

Descriptive data analysis was performed using SPSS version 25.^31^ Socio-demographic and clinical characteristics of participants were described using frequency counts and percentages. Mean and standard deviations (SD), median and inter-quartile range (IQR) were used to describe continuous variables. Confirmatory factor analysis was used to establish the construct validity of the PHQ-9 in the South African sample of 208 drug-susceptible TB patients. The models were fitted using lavaan version 0.5–23^32^ in R version 3.6.0.^33^ Confirmatory factor analysis requirements of multicollinearity, residual values, multivariate outliers and normality were examined. The data set satisfied the CFA assumptions of multicollinearity, residual values and multivariate outliers. However, the assumption of normality was violated for several variables. As variables were measured on a Likert scale and were thus ordinal rather than continuous, this is not surprising. In order to account for the violation of this assumption, the variables were specified as ‘ordered’ (ordinal variables) when fitting the CFA model. As the items were ordinal, a weighted least squares estimator with robust estimation of means and variances (WLSMV) was chosen. The comparative fit index (CFI) and the Tucker–Lewis Index (TLI) were used to determine whether the model fitted the data better compared to a more restricted baseline model. The root mean square error of approximation (RMSEA) was used to measure how closely the model represented data patterns. The model’s performance was tested by examining the differences between the expected and actual correlation matrix. The internal consistency of the PHQ-9 was evaluated by calculating Cronbach’s alpha.

### Ethical consideration

Participation in the research was voluntary. Trained bilingual fieldworkers explained the purpose of the study to eligible patients before the commencement of data collection. They were also informed about the voluntary nature of participation and assured about the anonymity of their information. Those who agreed to participate in the study provided verbal and written informed consent. Arrangements were in place to refer patients for further clinical assistance if they expressed distress during interviews. This study was approved by a Health Sciences Research Ethics Committee in the Free State province (UFS-HSD2019/1574/2611).

## Results

### Socio-demographic characteristics

The sample characteristics are displayed in Table 1. The majority of the patients in the study were male (*n* = 137; 65.9%). Patients’ median age was 38.5 (IQR: 30.3–54.0) years. The sample comprised largely of unmarried patients (*n* = 140; 67.3%) and patients who had attained secondary school education (*n* = 112; 53.8%). Almost 6 out of every 10 patients were co-infected with HIV (*n* = 118; 56.7%).

**TABLE 1:** Participant characteristics.

Variable	Total	
	*n*	%
**Sex**		
Male	137.0	65.9
Female	71.0	34.1
**Marital status**		
Married	68.0	32.7
Unmarried	140.0	67.3
**Education qualification**		
No formal education	3.0	1.4
Primary	53.0	25.5
Secondary	112.0	53.8
Matric (Grade 12)	34.0	16.4
Tertiary	6.0	2.9
**HIV status**		
Negative	80.0	38.5
Positive	118.0	56.7
Not recorded	10.0	4.8

HIV, human immunodeficiency virus; IQR, interquartile range.

Note: Age, Mean (SD) = 42.4 (15.2); Median (IQR) = 38.5 (30.3–54.00).

### Construct validity of the PHQ-9

Table 2 depicts the mean and SD of the nine items of the PHQ-9. Mean item scores ranged from 0.22 (SD = 0.61) to 1.14 (SD = 1.3).

**TABLE 2:** Descriptive characteristics for observed variables.

Variable	Mean	SD	Min.	Max.
1. Little interest or pleasure in doing things (anhedonia)	0.76	0.95	0	3
2. Feeling down, depressed or hopeless	0.80	0.90	0	3
3. Trouble falling or staying asleep (insomnia) or sleeping too much (hypersomnia)	0.92	1.14	0	3
4. Feeling tired or having little energy	0.99	1.11	0	3
5. Poor appetite or overeating	1.14	1.27	0	3
6. Feeling bad about yourself – or that you are a failure or have let yourself or your family down	0.51	0.88	0	3
7. Trouble concentrating on things such as reading the newspaper or watching television	0.47	0.83	0	3
8. Moving or speaking so slowly that other people could have noticed (retardation). Or the opposite – being so fidgety or restless that you have been moving around a lot more than usual (psychomotor agitation)	0.22	0.61	0	3
9. Thoughts that you would be better off dead or of hurting yourself in some way (suicidal ideation)	0.36	0.79	0	3

SD, standard deviation; Min., minimum; Max., maximum.

The model fit was good, with a TLI of 0.976, a CFI of 0.982 and an RMSEA of 0.062 (90% CI: 0.032–0.089). As expected, the indicators all showed significant positive factor loadings, with standardised coefficients ranging from 0.467 to 0.799 (Table 3). Taken together, these results are consistent with previous African studies showing a single factor latent structure for the PHQ-9, with the only latent factor underlying all items being depression. This finding confirms the hypothesis that the unidimensional structure of the PHQ-9 as presented in other clinical settings in Africa would be replicated in TB patients attending PHC facilities in the Free State province, and provides evidence for the construct validity of the PHQ-9. In terms of reliability, Cronbach’s alpha was 0.84, indicating that the PHQ-9 exhibited good internal consistency in this sample. The mean inter-item correlation was 0.37.

**TABLE 3:** Factor loadings.

Latent factor	Indicator	B	SE	Z	Beta	Sig.
Depression	Little interest or pleasure in doing things (anhedonia)	0.793	0.043	18.543	0.793	***
Depression	Feeling down, depressed or hopeless	0.751	0.047	15.978	0.751	***
Depression	Trouble falling or staying asleep (insomnia), or sleeping too much (hypersomnia)	0.729	0.050	14.693	0.729	***
Depression	Feeling tired or having little energy	0.799	0.038	20.802	0.799	***
Depression	Poor appetite or overeating	0.640	0.060	10.758	0.640	***
Depression	Feeling bad about yourself – or that you are a failure or have let yourself or your family down	0.643	0.059	10.966	0.643	***
Depression	Trouble concentrating on things such as reading the newspaper or watching television	0.788	0.045	17.366	0.788	***
Depression	Moving or speaking so slowly that other people could have noticed? Or the opposite – being so fidgety or restless that you have been moving around a lot more than usual	0.467	0.079	5.933	0.467	***
Depression	Thoughts that you would be better off dead or of hurting yourself in some way	0.721	0.063	11.478	0.721	***

B, unstandardised beta coefficient; SE, standard error; Z, standard Z-score; Beta, standardised beta coefficient; Sig, statistical significance.

## Discussion

The clarion call for the integration of mental health into PHC services worldwide reinforces the need to utilise psychometrically suitable assessment instruments. The PHQ-9 is widely used for assessing depression in PHC settings worldwide. This was the first time the tool was translated to Sesotho and used in the Free State province. As previous studies in clinical settings in Africa have employed EFA to establish the structure of the PHQ-9 for the assessment of depression,^24,28,34^ in this study, it was prudent to confirm the construct validity of the PHQ-9 using CFA. African studies have established variable cut-off points for probable depression, ranging from 5 to 11.^8,12,25,26^ Besides, the PHQ-9 is freely available, easy to understand and simple to score, making it even more advantageous to use in resource-constrained health settings, where administering comprehensive screening instruments may present challenges.^8^

The results confirmed the hypothesis that the unidimensional structure of the PHQ-9 established in other clinical settings in Africa^8,12,25^ would be replicated amongst TB patients attending PHC facilities in the Free State province, thereby providing evidence for the construct validity of the PHQ-9. The clinical implication of the unidimensional structure of the PHQ-9 is that the potential overlap between TB symptoms and somatic symptoms of depression may not significantly compromise the construct validity of the tool.^12^ Furthermore, clinicians can confidently count all symptoms towards the probable diagnosis of depression without necessarily having to scrutinise the aetiology of each symptom.^35^ Future studies in this Free State population should supplement the quantitative assessment with qualitative investigations on whether there are aspects that might require the adaptation of the PHQ-9 for culturally relevant use.

In this study, the PHQ-9 was forward-translated to Sesotho – the predominant local language amongst patients seeking PHC services in the Free State province – and back-translated to English by experienced translators. Previous research in Kenya finding a discrepancy between original and translated versions of the PHQ-9 highlighted challenges with the translation of the PHQ-9 because of differences between spoken or common and written or formal dialect and recommended stakeholder consensus on the translated tool.^36^ In this study, the translation of the questionnaire yielded minor discrepancies between the original and back-translated versions. These were duly discussed with the research team before consensus was reached on the final draft. Besides, reliability analysis showed that the internal consistency of the tool was satisfactory and was similar to other studies in Africa^8,12,25^ where the PHQ-9 was translated into local dialects. However, because of time and logistic limitations, it was not possible to administer the PHQ-9 twice to the TB patients to establish test–retest or inter-rater reliability.

The PHQ-9 in this study was interviewer-administered as opposed to being self-administered. Previous research has established consistent performance between self-administration and interviewer administration of the PHQ-9.^37^ A recent study whilst finding gaps in the assessment of common mental health disorders in adult patients attending PHC in KwaZulu-Natal province^38^ surmised that nurses might not have routinely identified these patients because of lack of clear pathways for treatment and follow-up. However, there is also a possibility that human resource constraints, such as inundation of professional health workers, negatively impacts the timely identification of patients with common mental health disorders such as depression.^39^ In light of extant professional health worker shortages in the Free State province,^40^ the TB programme could potentially explore the effect of supervised non-mental health specialists such as community health workers using this tool to screen and refer patients for clinical evaluation of depression. Ultimately, this would contribute to better TB treatment outcomes through early identification and treatment of depression in patients who might otherwise have been missed by the programme. A limitation of this study is that the sample was purposefully selected and results may not be generalised to other TB populations. Nonetheless, this study is the first to ascertain the construct validity of the PHQ-9 in a sample of susceptible TB patients in the Free State and South Africa.

## Conclusion

Results support the unidimensional structure of the PHQ-9, thereby confirming its construct validity. Besides, the tool has satisfactory internal consistency and is, therefore, reliable for use in the TB patient population. These findings provide TB programme coordinators with confidence in the psychometric properties of the PHQ-9 when considering its use in the routine mental health assessment of TB patients in the Free State province and similar settings.
